# The use of immersive technologies in learning about postpartum hemorrhage: Protocol for a systematic review and meta-analysis

**DOI:** 10.1371/journal.pone.0351749

**Published:** 2026-06-18

**Authors:** María Jose Montiel Bravo, Mariana Ferrandini Price, Manuel Pardo Rios, Marina Sanchez Gomez, Grasiela Piuvezam, Gidyenne Christine Bandeira Silva de Medeiros, Carmen Amalia Lopez Lopez

**Affiliations:** 1 Grupo de Investigación de Nuevas Tecnologías para la Salud, UCAM Universidad Católica de Murcia, Región de Murcia, Spain; 2 Facultad de Ciencias de la Salud, Universidad Católica de Murcia, Región de Murcia, Spain; 3 Hospital Universitario Rafael Méndez, Lorca, Región de Murcia, Spain; 4 Gerencia de Urgencias y Emergencias Sanitarias 061 de la, Región de Murcia, Spain; 5 Hospital Comarcal del Noroeste, Caravaca, Región de Murcia, Spain; 6 Postgraduation Program in Public Health (PPGSCOL), Federal University of Rio Grande do Norte, Natal, Brazil; 7 Systematic Review and Meta-Analysis Laboratory - Research & Health Innovation (Lab-SYS), CNPq-UFRN, Natal, Brazil; 8 Postgraduation Program in Health Sciences (PPGCSA), Federal University of Rio Grande do Norte, Natal, Brazil; Universidad Católica Sedes Sapientiae: Universidad Catolica Sedes Sapientiae, PERU

## Abstract

Postpartum hemorrhage is one of the leading causes of maternal morbidity and mortality worldwide, and its proper management requires both technical and non-technical skills. This study describes the protocol for a systematic review evaluating the effectiveness of immersive technologies in training healthcare professionals in the management of this obstetric emergency. This protocol was registered in the International Prospective Register of Systematic Reviews (PROSPERO) database (CRD420250614446). The search will be performed in the following databases: PubMed, Embase, Scopus, ScienceDirect, Web of Science, Cochrane Library, LILACS, Enfispo and CUIDEN. Intervention studies (clinical trials ‐ randomized or non-randomized) and quasi-experimental studies will be included. The risk of bias will be assessed using appropriate tools according to study design: the Risk of Bias 2 (ROB 2) tool for randomized controlled trials and the Risk Of Bias In Non-randomized Studies of Interventions (ROBINS-I) tool for non-randomized and quasi-experimental studies. Two independent researchers will conduct all assessments, and any disagreements will be consulted with a third reviewer. The data analysis and synthesis will be performed using Review Manager software version 5.4. We will conduct the study in accordance with the Preferred Reporting Items for Systematic Reviews and Meta-Analyses Protocols (PRISMA-P) guidelines. The review will summarize the current evidence on the use of virtual reality, augmented reality, and mixed reality in education and training for postpartum hemorrhage management. The planned systematic review will identify and synthesize the available evidence on how immersive technologies may improve learning about postpartum hemorrhage.

## Introduction

In the past decades, the field of patient safety has significantly evolved to become a core area of research and continuous improvement within health systems [1]. As a response to this challenge, immersive technologies, such as virtual reality (VR), augmented reality (AR), and mixed reality (MR), have acquired an increasingly important role in health education [2–4]. This is because these tools provide simulated, safe, and controlled environments that ease the development and refinement of clinical skills, allowing students to face complex scenarios without compromising the safety of patients [2–8]. The current evidence supports their usefulness, in particular for the acquisition of practical skills, the improvement in the making of clinical decisions, and the consolidation of knowledge [2,4–7].

In the area of obstetric training, immersive technologies are presented as especially promising resources. Their use in the teaching of critical maneuvers, such as basic life support in pregnant women, is associated with an increase in the confidence, and performance of students [7]. Likewise, their application in the training for the management of postpartum hemorrhage (PPH) has demonstrated significant improvements in key areas such as the management of tasks, teamwork, situational awareness, the making of decisions, and the satisfaction, motivation, and commitment of students as compared to traditional methods [9,10].

PPH has traditionally been defined as blood loss exceeding 500 mL after vaginal birth or 1000 mL following cesarean delivery [11,12]. However, this definition has evolved to include a more clinical perspective. In agreement with the most recent definition by the World Health Organization (WHO), PPH is characterized by an excessive loss of blood after the birth, which may lead to signs or symptoms of hypovolemia, regardless of the exact volume lost [13]. This approach recognizes that even minor losses can be clinically relevant if they compromise the hemodynamic stability of the patient, which reinforces the need for a comprehensive clinical assessment beyond the estimated quantification of blood loss [13].

PPH is still one of the main causes of maternal morbidity and mortality worldwide, with an incidence that oscillates between 5% and 10% of the births [10,14]. Its adequate management not only requires technical skills, but also non-technical skills such as effective communication, leadership, stress control, inter-professional collaboration, and situational awareness [9,15]. The literature suggests that a considerable portion of the adverse outcomes associated with PPH can be prevented with timely and adequate interventions, where simulation-based training plays a fundamental role [9,10,15].

Despite its clinical relevance, students’ exposure to real PPH cases is often limited, either due to the low frequency of these events in specific centers, or due to the predominantly observer role that they tend to play during these obstetric emergencies [5]. Faced with this reality, clinical simulations, especially in immersive environments, have become an efficient educational strategy to provide meaningful, practical, and safe training experiences [5,8,10,16]. Nevertheless, despite the advances described, doubts still exist about the long-term effectiveness of these technologies, as well as the methodological quality of the existing studies. In addition, aspects such as implementation costs, or the technical limitations, are relevant challenges for their large-scale implementation [2,4,8,10,17].

In this context, the aim of the present protocol is to describe the methodology of a systematic review oriented towards assessing the effectiveness of immersive technologies in the training of health professionals for addressing postpartum hemorrhage. The review intends to provide a critical and current synthesis of the available literature, in order to identify strengths, limitations, and areas for improvement in future educational interventions based on VR, AR, and MR.

## Materials and methods

### Study registration

The protocol for this systematic review was previously registered in the International Prospective Register of Systematic Reviews (PROSPERO), with registration number CRD420250614446. The development of the protocol followed the guidelines of the Cochrane Handbook for Systematic Reviews of Interventions [18] and the verification checklist for Systematic Reviews and Meta-Analysis Protocols (PRISMA-P) [19,20] (S1 File).

This protocol is based on the methodological standards previously established by our research group, with the relevant modifications for the present research question, ensuring reproducibility and alignment with international guidelines [21–23].

### Review question

The research question addressed in this review is: What are the learning outcomes of immersive technologies compared with traditional learning methods in terms of knowledge and learning experience related to postpartum hemorrhage?

### Eligibility criteria

- **Participants.** The studies included in the present review will involve health professionals and students in health-related fields.
- **Interventions.** The following studies will be considered: those that implement immersive technologies such as virtual reality, augmented reality, or 3D simulations to teach how to manage postpartum hemorrhage (PPH). The following will be excluded: studies in which the immersive technologies are only partially implemented or in which traditional simulations are used instead of completely immersive technologies, as the focus is to assess the overall impact of immersive technologies, and their partial use could affect the interpretation of the outcomes.
- **Comparison.** Studies with or without a control group will be included that compare the immersive technologies with traditional learning modalities. Traditional learning is characterized by a teacher-centered approach in which students are primarily passive recipients of information delivered by an instructor.
- **Outcomes.** The outcome measures will focus on:
  - Primary outcomes:
    - Knowledge about postpartum hemorrhage.
    - Satisfaction with the learning experience.
    - Technical skills (hard skills).
    - Non-technical skills (soft skills).
  - Secondary outcomes:
    - Adverse events, including reports of nausea and vomiting.

Studies that include training on obstetric emergencies that are different from postpartum hemorrhage will be excluded, unless they report specific outcomes related to postpartum hemorrhage management.

- **Types of studies.** Intervention studies will be included.

### Information sources and search strategies

Searches will be performed in the following databases: Scopus, MEDLINE (PubMed), Web of Science, EMBASE, ScienceDirect, Cochrane Library, LILACS, Enfispo, and CUIDEN.

For the search strategy, combinations of the following terms will be used: Medical Subject Headings (MeSH) (Students, Health Personnel, Virtual Reality, Augmented Reality, Computer Simulation, or Postpartum Hemorrhage, among others), as well as their synonyms (School Enrollment, Healthcare Workers, Simulation Training, Interactive Learning, Mixed Reality, among others), according to the characteristics of each database, in order to broaden the search coverage. In addition, to build the search equations, the terms will be combined with the Boolean operators “AND” and “OR”.

The specific search strategies will be available as supporting information in the S2 File.

No restrictions will be placed with respect to language or publication date.

### Study selection

All records retrieved from the database searches will be imported into Rayyan (version 0.1.0) to facilitate the management of references and the removal of duplicates. The screening process will be organized into two sequential stages.

Initially, two reviewers will independently screen titles and abstracts, discarding studies that do not fulfill the predefined eligibility criteria. Following this preliminary stage, the full texts of the remaining articles will be carefully examined to confirm their alignment with the established inclusion and exclusion parameters.

During the full-text assessment, the reference lists of the included studies will also be reviewed to detect any potentially relevant works that may have been missed during the initial database searches. Any disagreements will be resolved through discussion or consultation with a third reviewer.

To ensure transparency and reproducibility, the exclusion rationale for each discarded study will be recorded throughout the selection process. In addition, the inter-reviewer agreement in applying the eligibility criteria will be evaluated and documented.

### Data extraction and management

Data extraction will be performed independently by two reviewers. The data extracted will include study identification details (for example, aim, year, main author, country, and study design), the details of the intervention (for example, educational strategy, duration, and type of immersive technology utilized), the educational assessment measures and the reported outcomes (for example, knowledge about postpartum hemorrhage, and the learning experience of the participants).

Generative Artificial Intelligence (GAI) will be used only as a supportive tool to assist with the organization and preliminary structuring of data already extracted by the reviewers. Specifically, GAI may support the classification of extracted information into predefined categories, including study characteristics, participant profiles, type of immersive technology, intervention components, comparator characteristics, outcome domains, assessment instruments, time points, and reported adverse events. GAI may also assist in generating preliminary summary tables and identifying comparable outcome categories across studies.

GAI will not be used to determine study eligibility, perform independent data extraction, assess risk of bias, conduct GRADE assessment, calculate effect sizes, or make final methodological or interpretative decisions. All GAI-assisted outputs will be checked against the original full-text articles and the reviewers’ extraction forms by two independent human reviewers. Any discrepancy between the GAI-assisted output and the source data will be resolved through discussion, and, when necessary, by consultation with a third reviewer. The final validated dataset will be based exclusively on human-reviewed and verified information.

### Dealing with missing data

If relevant data are incomplete or unclear, the review team will attempt to obtain the missing information by contacting the study authors via email. When no response is received, the data will be excluded from the analysis. The missing data and their potential impact on the results will be reported in the discussion section.

### Risk of bias and certainty of evidence assessment

Randomized controlled trials will be assessed using Risk of Bias 2 (ROB 2), while non-randomized and quasi-experimental studies will be assessed using the Risk Of Bias In Non-randomized Studies of Interventions (ROBINS-I). The overall certainty of the evidence will be assessed using the Grading of Recommendations Assessment, Development and Evaluation (GRADE) approach.

Two reviewers will conduct the assessments independently, and any discrepancies will be resolved through discussion or consultation with a third reviewer.

### Data synthesis

Descriptive summaries and structured tables will be used to present the main characteristics and findings of the included studies.

Before considering quantitative synthesis, statistical heterogeneity will be examined using the chi-square (χ²) test with a significance threshold of p < 0.05, and the I² statistic will be calculated to estimate the proportion of variation across studies due to heterogeneity. An I² value exceeding 50% will be interpreted as substantial inconsistency.

When at least two studies demonstrate adequate clinical, methodological, and statistical similarity, a meta-analysis will be conducted using Review Manager (RevMan) version 5.4. For continuous outcomes, pooled effect sizes will be calculated using the standardized mean difference (SMD) with 95% confidence intervals. For dichotomous outcomes, effect sizes will be expressed as risk ratios (RR) with 95% confidence intervals.

A fixed-effect model will be applied when heterogeneity is low; otherwise, a random-effects model will be used. If substantial heterogeneity is identified and cannot be explained, only a narrative synthesis will be performed. Potential publication bias will be explored through funnel plots, and funnel plot asymmetry will be assessed using a regression-based method when sufficient studies are available.

During the narrative synthesis, GAI may be used to support the preliminary organization of findings by suggesting thematic groupings, identifying similarities across outcome domains, and drafting initial descriptive summaries based on the validated extraction tables. These AI-assisted summaries will be used only as preparatory material. The authors will verify all content against the original studies, revise the wording, confirm the accuracy of all classifications, and retain full responsibility for the final synthesis and interpretation of findings.

### Subgroup analyses

Subgroup analyses will be performed to explore possible variations in the learning outcomes as a function of the characteristics of the participants, the intervention, the comparator, and the study.

With respect to the participants, an analysis will be performed between health professionals and students in the related areas, as well as the level of education, distinguishing between undergraduate, postgraduate, and continuing education.

With respect to the intervention, the impact of the type of immersive technology used (virtual reality, augmented reality, or 3D simulations) will be assessed, as well as the duration and frequency of the intervention.

As for the comparator, the variations in the traditional learning methods used will be examined, such as masterclasses, seminars, or other approaches centered on the educators.

Lastly, the characteristics of the studies, including the sample size, the geographical region (high-income vs. middle- and low-income countries) will be analyzed, as well as the publication year, to identify possible variations in the results over time.

The heterogeneity within and between the sub-groups will be assessed with the I² statistic and the χ² test. If the data allow it, meta-regression analyses will be performed to examine the impact of these variables in the results. Otherwise, a narrative synthesis will be performed.

### Dissemination

The findings of the review will be shared with the scientific and healthcare communities through presentations at academic meetings, seminars, and other scholarly events. The findings will also be disseminated through peer-reviewed publications to promote knowledge transfer and support evidence-based practice.

## Discussion

Recent advances in immersive technologies are reshaping educational strategies in health sciences by enabling interactive and realistic learning environments that ensure patient safety [2]. Previous studies have reported generally positive outcomes in the development of non-technical competencies, including decision-making, teamwork, and situational awareness [9,15]. Improvements have also been noted in student satisfaction, engagement, and self-confidence [2,8,10,16]. However, some studies have found no statistically significant differences in these domains [5].

Regarding technical skills, the evidence is heterogeneous. While several investigations did not demonstrate superiority over conventional training methods [2,10], others identified clear benefits, particularly in specific contexts such as postpartum hemorrhage management [5,8,16]. Many authors emphasize that immersive technologies should be regarded as complementary educational tools, enhancing but not replacing traditional approaches [8,16].

A highly valued aspect in many of the studies was the capacity of the immersive technologies to overcome limitations in traditional teaching, such as the restricted access to real clinical situations or the passivity of students [4,5,8,15,16]. Simulation-based learning allows safe, repeatable practice that fosters student confidence and encourages active participation [5,8,15]. Nonetheless, the overall effectiveness of interventions based on immersive technologies interventions appears to depend on variables such as the degree of immersion, instructional quality, and feedback mechanisms implemented during the experience [8].

### Implications

PPH is a critical obstetric emergency that demands healthcare professionals to be proficient in both technical and non-technical skills [11–14]. However, opportunities for practical training in real clinical settings are often limited [5,9,10]. Within this context, immersive technologies have emerged as effective alternatives, providing controlled, realistic, and repeatable environments for learning without compromising patient safety [1,2,17].

This systematic review aims to generate comparative evidence on the educational outcomes achieved through training based on immersive technologies relative to traditional teaching approaches. The findings are expected to inform the design of more efficient, evidence-based educational programs that align with learners’ needs and support the development of health curricula.

Moreover, the review may contribute to the integration of immersive technologies into healthcare training frameworks, fostering improved preparedness among health professionals and ultimately enhancing the quality of maternal care.

### Limitations

This systematic review may encounter several limitations. Methodological heterogeneity, small sample sizes, lack of blinding, and limited long-term follow-up in the included studies could complicate comparisons across findings and potentially lower the overall quality of evidence according to the GRADE approach [2,4,17,24].

Minor adverse effects related to the use of immersive technologies, such as dizziness or nausea, may also restrict participants’ exposure time [2,4,9].

Additional structural and contextual challenges may include limited availability of technological resources, insufficient teacher training, and the digital divide between students and educators [4,8,17]. Furthermore, the limited participation of practicing healthcare professionals in many studies could reduce the external validity and applicability of the findings to real clinical environments [2,4,8,10,16].

Prior research, such as that by Kiesewetter et al. [25], has suggested that previous experience with technology may enhance learning outcomes, emphasizing the need for future studies that consider different levels of training and professional backgrounds [10,16,25].

## Supporting information

S1 File PRISMA-P checklist. (DOC)

S2 File Search strategies. (DOCX)
