# Supplementary material for: The use of immersive technologies in learning about postpartum hemorrhage: Protocol for a systematic review and meta-analysis
Source: PLoS One. 2026 Jun 18;21(6):e0351749. doi: 10.1371/journal.pone.0351749 (PMC13278411; doi:10.1371/journal.pone.0351749)
Supplement: S2 File — (DOCX) [file pone.0351749.s002.docx]

**S2 File. Search strategies.**

**PubMed/Medline search strategy.**

|  | **Search strategy** |
| --- | --- |
| **#1** | "Students"[Mesh] OR Student OR School Enrollment OR Enrollment, School OR Enrollments, School OR School Enrollments |
| **#2** | ("Health Personnel"[Mesh]) OR "Health Workforce"[Mesh] OR Personnel, Health OR Healthcare Workers OR Healthcare Worker OR Health Care Providers OR Health Care Provider OR Provider, Health Care OR Healthcare Providers OR Healthcare Provider OR Provider, Healthcare OR Health Care Professionals OR Health Care Professional OR Professional, Health Care |
| **#3** | **#1 OR #2** |
| **#4** | "Virtual Reality"[Mesh] OR Reality, Virtual OR Virtual Reality, Educational OR Educational Virtual Realities OR Educational Virtual Reality OR Reality, Educational Virtual OR Virtual Realities, Educational OR Virtual Reality, Instructional OR Instructional Virtual Realities OR Instructional Virtual Reality OR Realities, Instructional Virtual OR Reality, Instructional Virtual OR Virtual Realities, Instructional |
| **#5** | "Virtual Reality Exposure Therapy"[Mesh] OR Virtual Reality Immersion Therapy OR Virtual Reality Therapy OR Reality Therapies, Virtual OR Reality Therapy, Virtual OR Therapies, Virtual Reality OR Therapy, Virtual Reality OR Virtual Reality Therapies |
| **#6** | ("Augmented Reality"[Mesh]) OR "User-Computer Interface"[Mesh] OR Augmented Realities OR Realities, Augmented OR Reality, Augmented OR Mixed Reality OR Mixed Realities OR Realities, Mixed OR Reality, Mixed |
| **#7** | Simulation Training"[Mesh] OR Training, Simulation OR Interactive Learning OR Learning, Interactive |
| **#8** | "Computer Simulation"[Mesh] OR Computer Simulations OR Simulation, Computer OR Simulations, Computer OR Computer Models OR Computer Model OR Model, Computer OR In silico Models OR In silico Model OR Model, In silico OR In silico Simulation OR Simulation, In silico OR Computerized Models OR Computerized Model OR Model, Computerized OR Models, Computer OR Computational Modelling OR Modelling, Computational OR In silico Modeling OR Modeling, In silico OR Computational Modeling OR Modeling, Computational |
| **#9** | **#4 OR #5 OR #6 OR #7 OR #8** |
| **#10** | "Postpartum Hemorrhage"[Mesh] OR Hemorrhage, Postpartum OR Delayed Postpartum Hemorrhage OR Hemorrhage, Delayed Postpartum OR Postpartum Hemorrhage, Delayed OR Immediate Postpartum Hemorrhage OR Hemorrhage, Immediate Postpartum OR Postpartum Hemorrhage, Immediate |
| **#11** | **#3 AND #9 AND #10** |

**Cochrane search strategy.**

|  | **Search strategy** |
| --- | --- |
| **#1** | MeSH descriptor: [Students] explode all trees |
| **#2** | MeSH descriptor: [Health Personnel] explode all trees |
| **#3** | MeSH descriptor: [Health Workforce] explode all trees |
| **#4** | **#1 OR #2 OR #3** |
| **#5** | MeSH descriptor: [Virtual Reality] explode all trees |
| **#6** | MeSH descriptor: [Virtual Reality Exposure Therapy] explode all trees |
| **#7** | MeSH descriptor: [Augmented Reality] explode all trees |
| **#8** | MeSH descriptor: [User-Computer Interface] explode all trees |
| **#9** | MeSH descriptor: [Simulation Training] explode all trees |
| **#10** | MeSH descriptor: [High Fidelity Simulation Training] explode all trees |
| **#11** | MeSH descriptor: [Computer Simulation] explode all trees |
| **#12** | **#5 OR #6 OR #7 OR #8 OR #9 OR #10 OR #11** |
| **#13** | MeSH descriptor: [Postpartum Hemorrhage] explode all trees |
| **#14** | **#4 AND #12 AND #13** |

**Scopus search strategy.**

| **Search strategy** | TITLE-ABS-KEY ( ( ( Student OR Students OR "School Enrollment" OR "School Enrollments" ) OR ( "Health Personnel" OR "Health Workforce" OR "Healthcare Workers" OR "Healthcare Worker" OR "Health Care Providers" OR "Health Care Provider" OR "Health Care Professionals" OR "Health Care Professional" ) ) AND ( ( ( "Virtual Reality" OR "Virtual Reality, Educational" OR "Educational Virtual Reality" OR "Instructional Virtual Reality" OR "Instructional Virtual Realities" OR "Virtual Reality Exposure Therapy" OR "Virtual Reality Therapy" OR "Virtual Reality Immersion Therapy" OR "Virtual Reality Therapies" ) OR ( "Augmented Reality" OR "User-Computer Interface" OR "Mixed Reality" OR "Mixed Realities" ) OR ( "High Fidelity Simulation Training" OR "Simulation Training" OR "Interactive Learning" ) OR ( "Computer Simulation" OR "Computer Simulations" OR "In silico Models" OR "In silico Simulation" OR "Computational Modeling" OR "Computational Modelling" ) ) ) AND ( ( "Postpartum Hemorrhage" OR "Hemorrhage, Postpartum" OR "Delayed Postpartum Hemorrhage" OR "Immediate Postpartum Hemorrhage" ) ) ) |
| --- | --- |

**Web of Science search strategy.**

| **Search strategy** | TS=( ( (student* OR "school enrollment*" ) OR ("health personnel" OR "health workforce" OR "healthcare worker*" OR "health care provider*" OR "health care professional*") ) AND   ( ( ("virtual reality" OR "educational virtual reality" OR "instructional virtual reality" OR "virtual reality exposure therapy" OR "virtual reality therapy" OR "virtual reality immersion therapy" OR "virtual reality therapies") OR ("augmented reality" OR "user-computer interface" OR "mixed reality" OR "mixed realities") OR ("high fidelity simulation training" OR "simulation training" OR "interactive learning") OR ("computer simulation*" OR "in silico model*" OR "in silico simulation*" OR "computational modeling" OR "computational modelling") ) ) AND ( ("postpartum hemorrhage" OR "delayed postpartum hemorrhage" OR "immediate postpartum hemorrhage") ) ) |
| --- | --- |

**Embase search strategy.**

|  | **Search strategy** |
| --- | --- |
| **#1** | MeSH descriptor: [Students] explode all trees |
| **#2** | MeSH descriptor: [Health Personnel] explode all trees |
| **#3** | MeSH descriptor: [Health Workforce] explode all trees |
| **#4** | **#1 OR #2 OR #3** |
| **#5** | MeSH descriptor: [Virtual Reality] explode all trees |
| **#6** | MeSH descriptor: [Virtual Reality Exposure Therapy] explode all trees |
| **#7** | MeSH descriptor: [Augmented Reality] explode all trees |
| **#8** | MeSH descriptor: [User-Computer Interface] explode all trees |
| **#9** | MeSH descriptor: [Simulation Training] explode all trees |
| **#10** | MeSH descriptor: [High Fidelity Simulation Training] explode all trees |
| **#11** | MeSH descriptor: [Computer Simulation] explode all trees |
| **#12** | **#5 OR #6 OR #7 OR #8 OR #9 OR #10 OR #11** |
| **#13** | MeSH descriptor: [Postpartum Hemorrhage] explode all trees |
| **#14** | **#4 AND #12 AND #13** |

**Science Direct search strategy.**

| **Search strategy** | (Student OR "health personnel" OR "health care professional")AND("virtual reality" OR "augmented reality" OR "mixed reality" OR "simulation training" OR "computer simulation")AND("postpartum hemorrhage") |
| --- | --- |

**LILACS search strategy.**

| **Search strategy** | ("realidad virtual" OR "simulación virtual" OR "realidad aumentada" OR "tecnologías inmersivas" OR "simulación en pantalla" OR "entorno de aprendizaje de realidad virtual" OR "realidad mixta")  AND  ("hemorragia posparto") |
| --- | --- |

**ENFISPO search strategy.**

| **Search strategy** | ("realidad virtual" OR "simulación virtual" OR "realidad aumentada" OR "tecnologías inmersivas" OR "simulación en pantalla" OR "entorno de aprendizaje de realidad virtual" OR "realidad mixta")  AND  ("hemorragia posparto") |
| --- | --- |

**CUIDEN search strategy.**

| **Search strategy** | ("realidad virtual" OR "simulación virtual" OR "realidad aumentada" OR "tecnologías inmersivas" OR "simulación en pantalla" OR "entorno de aprendizaje de realidad virtual" OR "realidad mixta")  AND  ("hemorragia posparto") |
| --- | --- |
