# Supplementary material for: The use of immersive technologies in learning about postpartum hemorrhage: Protocol for a systematic review and meta-analysis
Source: PLoS One. 2026 Jun 18;21(6):e0351749. doi: 10.1371/journal.pone.0351749 (PMC13278411; doi:10.1371/journal.pone.0351749)
Supplement: Attachment — Submitted filename: R1. RESPONSE TO REVIEWERS.doc [file pone.0351749.s004.doc]

**Annisa Dewi Nugrahani**
Academic Editor
PLOS ONE

Dear Dr. Nugrahani,

We sincerely thank you and the reviewers for the careful evaluation of our manuscript entitled **“The use of immersive technologies in learning about postpartum hemorrhage: Protocol for a systematic review and meta-analysis”** (Manuscript ID: **PONE-D-25-57664**). We greatly appreciate the thoughtful and constructive feedback provided during the review process.

In response to the comments from the Academic Editor and reviewers, we have carefully revised the manuscript and addressed each point raised. We believe that these revisions have strengthened the clarity and quality of the manuscript.

Below, we provide a detailed, point-by-point response to all comments. All changes in the manuscript have been highlighted in the revised version with tracked changes.

We hope that our revisions meet the journal’s requirements and we would be pleased to provide any additional clarification or modifications if necessary.

Sincerely,

| **Reviewer Comment** | **Author Response** | **Page** |
| --- | --- | --- |
| **Journal Requirements** | | |
| **Formatting requirements**. Ensure that the manuscript meets PLOS ONE's style requirements, including file naming and formatting according to the PLOS ONE templates. | Thank you for this observation. The entire manuscript has been carefully reviewed and revised to ensure compliance with the PLOS ONE formatting and style requirements. File naming, manuscript structure, and formatting have been verified and adjusted according to the journal’s submission guidelines and templates. | All |
| **Funding inconsistency.** The grant information in the “Funding Information” and “Financial Disclosure” sections does not match. Please ensure the correct grant numbers are provided. | The funding statement has been updated accordingly in the revised manuscript. | 2 |
| **Role of funder statement.** Please state the role of the funders in the study. If the funders had no role, include the following statement:  "The funders had no role in study design, data collection and analysis, decision to publish, or preparation of the manuscript."  This statement should be included in the cover letter. | Thank you for this comment. The required statement regarding the role of the funders has been added to the the cover letter, as requested. | Cover  letter |
| **Funding in manuscript**. Funding information should not appear in the manuscript text. It should only appear in the Funding Statement section of the online submission form. | Thank you for this comment. The funding information has been removed from the manuscript text and will be provided exclusively in the Cover Letter and Funding Statement section of the online submission system, in accordance with PLOS ONE guidelines. | Cover  letter |
| **Data availability statement**. Please provide a complete Data Availability Statement in the submission form, including access information or justification if data cannot be made freely accessible. | Thank you for this comment. A complete Data Availability Statement has been provided in the submission form, indicating that no datasets were generated or analysed at this stage as the manuscript describes a systematic review protocol. The statement also clarifies that all relevant data will be made available in the final systematic review and its Supporting Information files upon study completion. | - |
| **Data sharing policy.** You indicated that data will be available upon acceptance. PLOS recommends deciding on a data sharing plan before acceptance and depositing the data in a public repository. If you cannot comply, please explain the reason. | Thank you for this comment. As this manuscript describes a protocol for a systematic review, no datasets have been generated at this stage. The Data Availability Statement in the submission form has been clarified to indicate that all relevant data extracted from the included studies will be made publicly available in the final publication and its Supporting Information files upon completion of the study. | - |
| **Supporting information captions.** Please include captions for Supporting Information files at the end of the manuscript and ensure that in-text citations match them. | Thank you for this comment. Captions for the Supporting Information files have been added at the end of the manuscript, and the in-text citations have been carefully checked to ensure they match the corresponding Supporting Information files. | 13 |
| **References suggested by reviewers.** If the reviewer comments include a recommendation to cite specific previously published works, please review and evaluate these publications to determine whether they are relevant and should be cited. There is no requirement to cite these works unless the editor has indicated otherwise. | We carefully reviewed the reviewer comments and note that no specific publications were suggested for citation. Therefore, no additional references were added. | - |
| **Editor's Comments** | | |
| **Statistical methods for meta-analysis.** Provide additional details about the statistical methods for the planned meta-analysis (e.g., effect size metrics, fixed vs random effects models). | Thank you for this comment. Additional details about the statistical methods planned for the meta-analysis have been incorporated into the Data Synthesis section of the manuscript. Specifically, we have clarified the effect size metrics, the statistical models to be used (fixed or random effects depending on heterogeneity), and the procedures for assessing heterogeneity and publication bias. | 8 |
| **Search strategy.** Expand the description of the search strategy or include complete search strings for several databases in the supplementary material. | We thank the reviewer for this helpful suggestion. The supplementary material containing the search strategy has been expanded to improve transparency and reproducibility. While the previous version included the search strategy for a single database, we have now added the complete search strategies for all databases included in the review. These are provided in S2 File. | S2 File |
| **Data repository.** Clarify the future data-sharing plan by specifying the repository where extracted data or review materials will be deposited. | Thank you for this comment. The future data-sharing plan has been clarified. The revised manuscript now specifies that the data extracted during the review process will be deposited in an open-access repository and will also be reported in the final publication and its Supporting Information files. | 9 |
| **Language editing.** Perform minor language editing to correct grammatical inconsistencies and improve readability. | Thank you for this comment. The manuscript has been carefully reviewed and minor language edits have been made throughout the text to improve grammar, clarity, and overall readability. | All |
