# Supplementary material for: The use of immersive technologies in learning about postpartum hemorrhage: Protocol for a systematic review and meta-analysis
Source: PLoS One. 2026 Jun 18;21(6):e0351749. doi: 10.1371/journal.pone.0351749 (PMC13278411; doi:10.1371/journal.pone.0351749)
Supplement: Attachment — Submitted filename: Response to Reviewers.doc [file pone.0351749.s005.doc]

**Annisa Dewi Nugrahani**
Academic Editor
PLOS ONE

Dear Dr. Nugrahani,

We sincerely thank you and the reviewers for the careful evaluation of our manuscript entitled **“The use of immersive technologies in learning about postpartum hemorrhage: Protocol for a systematic review and meta-analysis”** (Manuscript ID: **PON-D-25-57664R1**). We greatly appreciate the thoughtful and constructive feedback provided during the review process.

In response to the comments from the Academic Editor and reviewers, we have carefully revised the manuscript and addressed each point raised. We believe that these revisions have strengthened the clarity and quality of the manuscript.

Below, we provide a detailed, point-by-point response to all comments. All changes in the manuscript have been highlighted in the revised version with tracked changes.

We hope that our revisions meet the journal’s requirements and we would be pleased to provide any additional clarification or modifications if necessary.

Sincerely,

| **Reviewer** | **Author Response** | **Page** |
| --- | --- | --- |
| **Reviewer 1** | | |
| **Correction of minor typographical and formatting issues** | Thank you for this observation. The manuscript has been carefully proofread in full. Minor typographical and formatting issues have been corrected throughout the text, including spacing inconsistencies, incomplete sentences, punctuation, superscript formatting in the author affiliations, capitalization, hyphenation, and reference-list formatting. We also revised minor wording issues to improve clarity and consistency without modifying the scientific content of the manuscript. | All |
| **Consistency in terminology.**  Ensure uniform use of key terms throughout the manuscript, including: – outcomes / results  – immersive technologies  – consistent naming of methodological tools (ROB 2, ROBINS‑I, GRADE) | Thank you for this helpful comment. We have revised the manuscript to ensure consistent terminology throughout. Specifically, we standardized the use of “outcomes” when referring to the variables and endpoints assessed in the review, and we reserved “findings” or “results” for the evidence reported by included studies or for the final outputs of the review. We also harmonized the terminology related to immersive technologies throughout the manuscript and avoided inconsistent alternation with abbreviated or less precise expressions. Finally, we standardized the names of the methodological tools as Risk of Bias 2 (ROB 2), Risk Of Bias In Non-randomized Studies of Interventions (ROBINS-I), and Grading of Recommendations Assessment, Development and Evaluation (GRADE). These changes have been implemented across the Abstract, Eligibility criteria, Data extraction and management, Risk of bias and certainty of evidence assessment, Data synthesis, Discussion, Implications, and Limitations sections. | All |
| **Clarification of AI use.**  The manuscript describes the incorporation of Generative Artificial Intelligence (GAI) to support the organization and preliminary structuring of extracted data. Reviewer 1 notes that this description remains general. Please provide a brief clarification specifying the collaborative human–AI workflow in greater detail (i.e., what aspects of data organization the GAI will support and how human reviewers will oversee and validate these steps). | Thank you for this helpful comment. We have revised the “Data extraction and management” and “Data synthesis” sections to clarify the planned human–GAI collaborative workflow. We now specify that GAI will be used only as a supportive tool to assist with the preliminary organization and structuring of data already extracted by human reviewers. In particular, GAI may support the classification of extracted information into predefined categories, including study characteristics, participant profiles, type of immersive technology, intervention components, comparator characteristics, outcome domains, assessment instruments, time points, and reported adverse events. It may also assist in generating preliminary summary tables and identifying comparable outcome categories across studies.  We have also clarified that GAI will not be used to determine study eligibility, perform independent data extraction, assess risk of bias, conduct GRADE assessment, calculate effect sizes, or make final methodological or interpretative decisions. All GAI-assisted outputs will be checked against the original full-text articles and the reviewers’ extraction forms by two independent human reviewers. Any discrepancies will be resolved through discussion or, when necessary, by consultation with a third reviewer. The final dataset, synthesis, and interpretation will remain the sole responsibility of the review team. | All |
