# Supplementary material for: The use of immersive technologies in learning about postpartum hemorrhage: Protocol for a systematic review and meta-analysis
Source: PLoS One. 2026 Jun 18;21(6):e0351749. doi: 10.1371/journal.pone.0351749 (PMC13278411; doi:10.1371/journal.pone.0351749)
Supplement: Peer review history

# Peer review: Decision Letter 0

**DOI:** 10.1371/journal.pone.0351749.r001

**Authors:** Annisa Nugrahani

5 Mar 2026

PONE-D-25-57664

The use of immersive technologies in learning about postpartum hemorrhage: Protocol for a systematic review and meta-analysis

PLOS One

Dear Dr. Rios,

Thank you for submitting your manuscript to PLOS ONE. After careful consideration, we feel that it has merit but does not fully meet PLOS ONE’s publication criteria as it currently stands. Therefore, we invite you to submit a revised version of the manuscript that addresses the points raised during the review process.

Please submit your revised manuscript by Apr 19 2026 11:59PM. If you will need more time than this to complete your revisions, please reply to this message or contact the journal office at plosone@plos.org. When you're ready to submit your revision, log on to https://www.editorialmanager.com/pone/ and select the 'Submissions Needing Revision' folder to locate your manuscript file.

Please include the following items when submitting your revised manuscript:

- A letter that responds to each point raised by the academic editor and reviewer(s). You should upload this letter as a separate file labeled 'Response to Reviewers'.
- A marked-up copy of your manuscript that highlights changes made to the original version. You should upload this as a separate file labeled 'Revised Manuscript with Track Changes'.
- An unmarked version of your revised paper without tracked changes. You should upload this as a separate file labeled 'Manuscript'.

If you would like to make changes to your financial disclosure, please include your updated statement in your cover letter. Guidelines for resubmitting your figure files are available below the reviewer comments at the end of this letter.

If applicable, we recommend that you deposit your laboratory protocols in protocols.io to enhance the reproducibility of your results. Protocols.io assigns your protocol its own identifier (DOI) so that it can be cited independently in the future. For instructions see: https://journals.plos.org/plosone/s/submission-guidelines#loc-laboratory-protocols. Additionally, PLOS ONE offers an option for publishing peer-reviewed Lab Protocol articles, which describe protocols hosted on protocols.io. Read more information on sharing protocols at https://plos.org/protocols?utm_medium=editorial-email&utm_source=authorletters&utm_campaign=protocols.

We look forward to receiving your revised manuscript.

Kind regards,

Annisa Dewi Nugrahani

Academic Editor

PLOS One

Journal Requirements:

When submitting your revision, we need you to address these additional requirements.

1.Please ensure that your manuscript meets PLOS ONE's style requirements, including those for file naming. The PLOS ONE style templates can be found at

https://journals.plos.org/plosone/s/file?id=wjVg/PLOSOne_formatting_sample_main_body.pdf and

https://journals.plos.org/plosone/s/file?id=ba62/PLOSOne_formatting_sample_title_authors_affiliations.pdf

2. We note that the grant information you provided in the ‘Funding Information’ and ‘Financial Disclosure’ sections do not match.

When you resubmit, please ensure that you provide the correct grant numbers for the awards you received for your study in the ‘Funding Information’ section.

3. Thank you for stating the following financial disclosure:

“This work was funded by the Ministry of Science, Innovation and Universities in the research project entitled Diseño de un Metaverso para la Educación Superior en Ciencias de la Salud  (Designing a Metaverse for Higher Education in Health Sciences) (PID2022-138884NA-I00), reference MCIN/AEI/10.13039/501100011033/FEDER, UE.”

Please state what role the funders took in the study.  If the funders had no role, please state: "The funders had no role in study design, data collection and analysis, decision to publish, or preparation of the manuscript."

If this statement is not correct you must amend it as needed.

Please include this amended Role of Funder statement in your cover letter; we will change the online submission form on your behalf.

4. Please note that funding information should not appear in any section or other areas of your manuscript. We will only publish funding information present in the Funding Statement section of the online submission form. Please remove any funding-related text from the manuscript.

5. Please provide a complete Data Availability Statement in the submission form, ensuring you include all necessary access information or a reason for why you are unable to make your data freely accessible. If your research concerns only data provided within your submission, please write "All data are in the manuscript and/or supporting information files" as your Data Availability Statement.

6. When completing the data availability statement of the submission form, you indicated that you will make your data available on acceptance. We strongly recommend all authors decide on a data sharing plan before acceptance, as the process can be lengthy and hold up publication timelines. Please note that, though access restrictions are acceptable now, your entire data will need to be made freely accessible if your manuscript is accepted for publication. This policy applies to all data except where public deposition would breach compliance with the protocol approved by your research ethics board. If you are unable to adhere to our open data policy, please kindly revise your statement to explain your reasoning and we will seek the editor's input on an exemption. Please be assured that, once you have provided your new statement, the assessment of your exemption will not hold up the peer review process.

7. Please include captions for your Supporting Information files at the end of your manuscript, and update any in-text citations to match accordingly. Please see our Supporting Information guidelines for more information: http://journals.plos.org/plosone/s/supporting-information.

8. If the reviewer comments include a recommendation to cite specific previously published works, please review and evaluate these publications to determine whether they are relevant and should be cited. There is no requirement to cite these works unless the editor has indicated otherwise.

[Note: HTML markup is below. Please do not edit.]

Reviewer's Responses to Questions

**Comments to the Author**

1. Does the manuscript provide a valid rationale for the proposed study, with clearly identified and justified research questions?

The research question outlined is expected to address a valid academic problem or topic and contribute to the base of knowledge in the field.

Reviewer #1: Yes

**********

2. Is the protocol technically sound and planned in a manner that will lead to a meaningful outcome and allow testing the stated hypotheses?

The manuscript should describe the methods in sufficient detail to prevent undisclosed flexibility in the experimental procedure or analysis pipeline, including sufficient outcome-neutral conditions (e.g. necessary controls, absence of floor or ceiling effects) to test the proposed hypotheses and a statistical power analysis where applicable. As there may be aspects of the methodology and analysis which can only be refined once the work is undertaken, authors should outline potential assumptions and explicitly describe what aspects of the proposed analyses, if any, are exploratory.

Reviewer #1: Partly

**********

3. Is the methodology feasible and described in sufficient detail to allow the work to be replicable?

Descriptions of methods and materials in the protocol should be reported in sufficient detail for another researcher to reproduce all experiments and analyses. The protocol should describe the appropriate controls, sample size calculations, and replication needed to ensure that the data are robust and reproducible.

Reviewer #1: Yes

**********

4. Have the authors described where all data underlying the findings will be made available when the study is complete?

The PLOS Data policy requires authors to make all data underlying the findings described in their manuscript fully available without restriction, with rare exception, at the time of publication. The data should be provided as part of the manuscript or its supporting information, or deposited to a public repository. For example, in addition to summary statistics, the data points behind means, medians and variance measures should be available. If there are restrictions on publicly sharing data—e.g. participant privacy or use of data from a third party—those must be specified.

Reviewer #1: No

**********

5. Is the manuscript presented in an intelligible fashion and written in standard English?

PLOS ONE does not copyedit accepted manuscripts, so the language in submitted articles must be clear, correct, and unambiguous. Any typographical or grammatical errors should be corrected at revision, so please note any specific errors here.

Reviewer #1: Yes

**********

6. Review Comments to the Author

Please use the space provided to explain your answers to the questions above and, if applicable, provide comments about issues authors must address before this protocol can be accepted for publication. You may also include additional comments for the author, including concerns about research or publication ethics.

You may also provide optional suggestions and comments to authors that they might find helpful in planning their study.

(Please upload your review as an attachment if it exceeds 20,000 characters)

Reviewer #1: To further strengthen the manuscript, the authors may consider:

Providing additional details about the statistical methods for the planned meta-analysis (e.g., effect size metrics, fixed vs random effects models).

Expanding the description of the search strategy or including complete search strings for several databases in supplementary material.

Clarifying the future data-sharing plan by specifying the repository where extracted data or review materials will be deposited.

Performing minor language editing to correct grammatical inconsistencies and improve readability.

**********

7. PLOS authors have the option to publish the peer review history of their article (what does this mean?). If published, this will include your full peer review and any attached files.

If you choose “no”, your identity will remain anonymous but your review may still be made public.

**Do you want your identity to be public for this peer review?**  For information about this choice, including consent withdrawal, please see our Privacy Policy.

Reviewer #1: No

**********

[NOTE: If reviewer comments were submitted as an attachment file, they will be attached to this email and accessible via the submission site. Please log into your account, locate the manuscript record, and check for the action link "View Attachments". If this link does not appear, there are no attachment files.]

To ensure your figures meet our technical requirements, please review our figure guidelines: https://journals.plos.org/plosone/s/figures

You may also use PLOS’s free figure tool, NAAS, to help you prepare publication quality figures: https://journals.plos.org/plosone/s/figures#loc-tools-for-figure-preparation.

NAAS will assess whether your figures meet our technical requirements by comparing each figure against our figure specifications.

# Author comment: Author response to Decision Letter 1

**DOI:** 10.1371/journal.pone.0351749.r002

8 Mar 2026

The response to the reviewers has been uploaded as a separate file.

Attachment Submitted filename: R1. RESPONSE TO REVIEWERS.doc

# Peer review: Decision Letter 1

**DOI:** 10.1371/journal.pone.0351749.r003

**Authors:** Yordanis Enríquez Canto

30 Apr 2026

PONE-D-25-57664R1

The use of immersive technologies in learning about postpartum hemorrhage: Protocol for a systematic review and meta-analysis

PLOS One

Dear Dr. Rios,

Thank you for submitting your manuscript to PLOS ONE. After careful consideration, we feel that it has merit but does not fully meet PLOS ONE’s publication criteria as it currently stands. Therefore, we invite you to submit a revised version of the manuscript that addresses the points raised during the review process.

I am pleased to note that the scientific content and methodological structure of the protocol are sound, and the reviewer considers the manuscript suitable for publication pending minor revisions. These refinements do not affect the validity of the protocol but are necessary to ensure clarity, consistency, and full alignment with journal standards. Once these minor issues are addressed, the manuscript will be ready for acceptance.

Please submit your revised manuscript by Jun 13 2026 11:59PM. If you will need more time than this to complete your revisions, please reply to this message or contact the journal office at plosone@plos.org. When you're ready to submit your revision, log on to https://www.editorialmanager.com/pone/ and select the 'Submissions Needing Revision' folder to locate your manuscript file.

Please include the following items when submitting your revised manuscript:

- A letter that responds to each point raised by the academic editor and reviewer(s). You should upload this letter as a separate file labeled 'Response to Reviewers'.
- A marked-up copy of your manuscript that highlights changes made to the original version. You should upload this as a separate file labeled 'Revised Manuscript with Track Changes'.
- An unmarked version of your revised paper without tracked changes. You should upload this as a separate file labeled 'Manuscript'.

If you would like to make changes to your financial disclosure, please include your updated statement in your cover letter. Guidelines for resubmitting your figure files are available below the reviewer comments at the end of this letter.

If applicable, we recommend that you deposit your laboratory protocols in protocols.io to enhance the reproducibility of your results. Protocols.io assigns your protocol its own identifier (DOI) so that it can be cited independently in the future. For instructions see: https://journals.plos.org/plosone/s/submission-guidelines#loc-laboratory-protocols. Additionally, PLOS ONE offers an option for publishing peer-reviewed Lab Protocol articles, which describe protocols hosted on protocols.io. Read more information on sharing protocols at https://plos.org/protocols?utm_medium=editorial-email&utm_source=authorletters&utm_campaign=protocols.

As the corresponding author, your ORCID iD is verified in the submission system and will appear in the published article. PLOS supports the use of ORCID, and we encourage all coauthors to register for an ORCID iD and use it as well. Please encourage your coauthors to verify their ORCID iD within the submission system before final acceptance, as unverified ORCID iDs will not appear in the published article. *Only*  the individual author can complete the verification step; PLOS staff *cannot*  verify ORCID iDs on behalf of authors.

We look forward to receiving your revised manuscript.

Kind regards,

Yordanis Enríquez Canto, Ph.D.

Academic Editor

PLOS One

Journal Requirements:

If the reviewer comments include a recommendation to cite specific previously published works, please review and evaluate these publications to determine whether they are relevant and should be cited. There is no requirement to cite these works unless the editor has indicated otherwise.

Please review your reference list to ensure that it is complete and correct. If you have cited papers that have been retracted, please include the rationale for doing so in the manuscript text, or remove these references and replace them with relevant current references. Any changes to the reference list should be mentioned in the rebuttal letter that accompanies your revised manuscript. If you need to cite a retracted article, indicate the article’s retracted status in the References list and also include a citation and full reference for the retraction notice.

Additional Editor Comments:

Please address the following points in your next revision:

Correction of minor typographical and formatting issues

Reviewer 1 notes that some duplicated words, spacing inconsistencies, and formatting artifacts remain. Please carefully proofread the full manuscript to ensure it is fully polished.

Consistency in terminology

Ensure uniform use of key terms throughout the manuscript, including:

– outcomes / results

– immersive technologies

– consistent naming of methodological tools (ROB 2, ROBINS‑I, GRADE)

Clarification of AI use

The manuscript describes the incorporation of Generative Artificial Intelligence (GAI) to support the organization and preliminary structuring of extracted data. Reviewer 1 notes that this description remains general. Please provide a brief clarification specifying the collaborative human–AI workflow in greater detail (i.e., what aspects of data organization the GAI will support and how human reviewers will oversee and validate these steps).

[Note: HTML markup is below. Please do not edit.]

Reviewers' comments:

Reviewer's Responses to Questions

**Comments to the Author**

1. Does the manuscript provide a valid rationale for the proposed study, with clearly identified and justified research questions?

The research question outlined is expected to address a valid academic problem or topic and contribute to the base of knowledge in the field.

Reviewer #1: Yes

**********

2. Is the protocol technically sound and planned in a manner that will lead to a meaningful outcome and allow testing the stated hypotheses?

The manuscript should describe the methods in sufficient detail to prevent undisclosed flexibility in the experimental procedure or analysis pipeline, including sufficient outcome-neutral conditions (e.g. necessary controls, absence of floor or ceiling effects) to test the proposed hypotheses and a statistical power analysis where applicable. As there may be aspects of the methodology and analysis which can only be refined once the work is undertaken, authors should outline potential assumptions and explicitly describe what aspects of the proposed analyses, if any, are exploratory.

Reviewer #1: Yes

**********

3. Is the methodology feasible and described in sufficient detail to allow the work to be replicable?

Descriptions of methods and materials in the protocol should be reported in sufficient detail for another researcher to reproduce all experiments and analyses. The protocol should describe the appropriate controls, sample size calculations, and replication needed to ensure that the data are robust and reproducible.

Reviewer #1: Yes

**********

4. Have the authors described where all data underlying the findings will be made available when the study is complete?

The PLOS Data policy requires authors to make all data underlying the findings described in their manuscript fully available without restriction, with rare exception, at the time of publication. The data should be provided as part of the manuscript or its supporting information, or deposited to a public repository. For example, in addition to summary statistics, the data points behind means, medians and variance measures should be available. If there are restrictions on publicly sharing data—e.g. participant privacy or use of data from a third party—those must be specified.

Reviewer #1: Yes

**********

5. Is the manuscript presented in an intelligible fashion and written in standard English?

PLOS ONE does not copyedit accepted manuscripts, so the language in submitted articles must be clear, correct, and unambiguous. Any typographical or grammatical errors should be corrected at revision, so please note any specific errors here.

Reviewer #1: Yes

**********

6. Review Comments to the Author

Please use the space provided to explain your answers to the questions above and, if applicable, provide comments about issues authors must address before this protocol can be accepted for publication. You may also include additional comments for the author, including concerns about research or publication ethics.

You may also provide optional suggestions and comments to authors that they might find helpful in planning their study.

(Please upload your review as an attachment if it exceeds 20,000 characters)

Reviewer #1: A few minor issues should be addressed before final acceptance:

Some minor inconsistencies and typographical errors remain (e.g., duplicated words, formatting issues in the revised version). Please ensure the final version is fully cleaned.

Consistency in terminology

Ensure consistent use of terms such as outcomes, results, immersive technologies, and naming of tools (ROB 2, ROBINS-I, GRADE) throughout the manuscript.

Clarification of AI use

Overall, these are minor refinements and do not affect the scientific quality of the protocol.

**********

7. PLOS authors have the option to publish the peer review history of their article (what does this mean?). If published, this will include your full peer review and any attached files.

If you choose “no”, your identity will remain anonymous but your review may still be made public.

**Do you want your identity to be public for this peer review?**  For information about this choice, including consent withdrawal, please see our Privacy Policy.

Reviewer #1: No

**********

[NOTE: If reviewer comments were submitted as an attachment file, they will be attached to this email and accessible via the submission site. Please log into your account, locate the manuscript record, and check for the action link "View Attachments". If this link does not appear, there are no attachment files.]

To ensure your figures meet our technical requirements, please review our figure guidelines: https://journals.plos.org/plosone/s/figures

You may also use PLOS’s free figure tool, NAAS, to help you prepare publication quality figures: https://journals.plos.org/plosone/s/figures#loc-tools-for-figure-preparation.

NAAS will assess whether your figures meet our technical requirements by comparing each figure against our figure specifications.

# Author comment: Author response to Decision Letter 2

**DOI:** 10.1371/journal.pone.0351749.r004

1 May 2026

Dear Dr. Nugrahani,

We sincerely thank you and the reviewer for the careful evaluation of our manuscript entitled “The use of immersive technologies in learning about postpartum hemorrhage: Protocol for a systematic review and meta-analysis” (Manuscript ID: PON-D-25-57664R1). We greatly appreciate the thoughtful and constructive feedback provided during the review process.

In response to the comments from the Academic Editor and Reviewer 1, we have carefully revised the manuscript and addressed each point raised. All changes have been incorporated into the revised manuscript with tracked changes. We believe that these revisions have strengthened the clarity, consistency, and quality of the manuscript.

Reviewer 1

Comment 1: Correction of minor typographical and formatting issues.

Response: Thank you for this observation. The manuscript has been carefully proofread in full. Minor typographical and formatting issues have been corrected throughout the text, including spacing inconsistencies, incomplete sentences, punctuation, superscript formatting in the author affiliations, capitalization, hyphenation, and reference-list formatting. We also revised minor wording issues to improve clarity and consistency without modifying the scientific content of the manuscript.

Comment 2: Consistency in terminology. Ensure uniform use of key terms throughout the manuscript, including outcomes/results, immersive technologies, and consistent naming of methodological tools (ROB 2, ROBINS-I, GRADE).

Response: Thank you for this helpful comment. We have revised the manuscript to ensure consistent terminology throughout. Specifically, we standardized the use of “outcomes” when referring to the variables and endpoints assessed in the review, and we reserved “findings” or “results” for the evidence reported by included studies or for the final outputs of the review. We also harmonized the terminology related to immersive technologies throughout the manuscript and avoided inconsistent alternation with abbreviated or less precise expressions. Finally, we standardized the names of the methodological tools as Risk of Bias 2 (ROB 2), Risk Of Bias In Non-randomized Studies of Interventions (ROBINS-I), and Grading of Recommendations Assessment, Development and Evaluation (GRADE). These changes have been implemented across the Abstract, Eligibility criteria, Data extraction and management, Risk of bias and certainty of evidence assessment, Data synthesis, Discussion, Implications, and Limitations sections.

Comment 3: Clarification of AI use. The manuscript describes the incorporation of Generative Artificial Intelligence (GAI) to support the organization and preliminary structuring of extracted data. Reviewer 1 notes that this description remains general. Please provide a brief clarification specifying the collaborative human–AI workflow in greater detail, including what aspects of data organization the GAI will support and how human reviewers will oversee and validate these steps.

Response: Thank you for this helpful comment. We have revised the “Data extraction and management” and “Data synthesis” sections to clarify the planned human–GAI collaborative workflow. We now specify that GAI will be used only as a supportive tool to assist with the preliminary organization and structuring of data already extracted by human reviewers. In particular, GAI may support the classification of extracted information into predefined categories, including study characteristics, participant profiles, type of immersive technology, intervention components, comparator characteristics, outcome domains, assessment instruments, time points, and reported adverse events. It may also assist in generating preliminary summary tables and identifying comparable outcome categories across studies.

We have also clarified that GAI will not be used to determine study eligibility, perform independent data extraction, assess risk of bias, conduct GRADE assessment, calculate effect sizes, or make final methodological or interpretative decisions. All GAI-assisted outputs will be checked against the original full-text articles and the reviewers’ extraction forms by two independent human reviewers. Any discrepancies will be resolved through discussion or, when necessary, by consultation with a third reviewer. The final dataset, synthesis, and interpretation will remain the sole responsibility of the review team.

We hope that our revisions meet the journal’s requirements and remain available to provide any additional clarification if necessary.

Sincerely,

The authors

Attachment Submitted filename: Response to Reviewers.doc

# Editor report: Decision Letter 2

**DOI:** 10.1371/journal.pone.0351749.r005

**Authors:** Yordanis Enríquez Canto

2 Jun 2026

The use of immersive technologies in learning about postpartum hemorrhage: Protocol for a systematic review and meta-analysis

PONE-D-25-57664R2

Dear Dr. Rios,

We’re pleased to inform you that your manuscript has been judged scientifically suitable for publication and will be formally accepted for publication once it meets all outstanding technical requirements.

Within one week, you’ll receive an e-mail detailing the required amendments. When these have been addressed, you’ll receive a formal acceptance letter and your manuscript will be scheduled for publication.

An invoice will be generated when your article is formally accepted. Please note, if your institution has a publishing partnership with PLOS and your article meets the relevant criteria, all or part of your publication costs will be covered. Please make sure your user information is up-to-date by logging into Editorial Manager at Editorial Manager® and clicking the ‘Update My Information' link at the top of the page. For questions related to billing, please contact billing support.

If your institution or institutions have a press office, please notify them about your upcoming paper to help maximize its impact. If they’ll be preparing press materials, please inform our press team as soon as possible -- no later than 48 hours after receiving the formal acceptance. Your manuscript will remain under strict press embargo until 2 pm Eastern Time on the date of publication. For more information, please contact onepress@plos.org.

Kind regards,

Yordanis Enríquez Canto, Ph.D.

Academic Editor

PLOS One

Additional Editor Comments (optional):

Reviewers' comments:

# Editor report: Acceptance letter

**DOI:** 10.1371/journal.pone.0351749.r006

**Authors:** Yordanis Enríquez Canto

PONE-D-25-57664R2

PLOS One

Dear Dr. Rios,

I'm pleased to inform you that your manuscript has been deemed suitable for publication in PLOS One. Congratulations! Your manuscript is now being handed over to our production team.

At this stage, our production department will prepare your paper for publication. This includes ensuring the following:

* All references, tables, and figures are properly cited

* All relevant supporting information is included in the manuscript submission,

* There are no issues that prevent the paper from being properly typeset

You will receive further instructions from the production team, including instructions on how to review your proof when it is ready. Please keep in mind that we are working through a large volume of accepted articles, so please give us a few days to review your paper and let you know the next and final steps.

Lastly, if your institution or institutions have a press office, please let them know about your upcoming paper now to help maximize its impact. If they'll be preparing press materials, please inform our press team within the next 48 hours. Your manuscript will remain under strict press embargo until 2 pm Eastern Time on the date of publication. For more information, please contact onepress@plos.org.

You will receive an invoice from PLOS for your publication fee after your manuscript has reached the completed accept phase. If you receive an email requesting payment before acceptance or for any other service, this may be a phishing scheme. Learn how to identify phishing emails and protect your accounts at https://explore.plos.org/phishing.

If we can help with anything else, please email us at customercare@plos.org.

Thank you for submitting your work to PLOS ONE and supporting open access.

Kind regards,

PLOS ONE Editorial Office Staff

on behalf of

Prof. Yordanis Enríquez Canto

Academic Editor

PLOS One
